# Short and longterm outcome of minimally invasive therapy of median arcuate ligament syndrome

**DOI:** 10.1007/s00423-024-03511-9

**Published:** 2024-10-24

**Authors:** Frederike Butz, Oliver Haase, Friederike Martin, Karl Herbert Hillebrandt, Sebastian Knitter, Wenzel Schöning, Nathanael Raschzok, Johann Pratschke, Felix Krenzien

**Affiliations:** 1 Department of Surgery, Campus Charité Mitte| Campus Virchow-Klinikum, Charité– Universitätsmedizin Berlin, Corporate Member of Freie Universität Berlin, Humboldt-Universität Zu Berlin, Augustenburger Platz 1, 13353 Berlin, Germany; 2 Berlin Institute of Health at Charité - Universitätsmedizin Berlin, BIH Biomedical Innovation Academy, BIH Charité Clinician Scientist Program, Berlin, Germany

**Keywords:** Median arcuate ligament syndrome, Robotic-assisted surgery, Minimally invasive surgery

## Abstract

**Purpose:**

Median arcuate ligament syndrome (MALS) is a rare disorder caused by compression of the celiac artery (CA) by the median arcuate ligament (MAL). Common symptoms include postprandial abdominal pain, diarrhea, and weight loss. While laparoscopic MAL division has long been considered the procedure of choice, robotic-assisted procedures have been increasingly used since their introduction. Aim of this study was to evaluate peri- and postoperative outcomes after minimally invasive MAL release.

**Methods:**

A retrospective analysis of patients undergoing minimally invasive MAL release at the Department of Surgery, Charité - Universitätsmedizin Berlin, between 2014 and 2023 was performed.

**Results:**

20 patients met the inclusion criteria and underwent either laparoscopic (*n* = 3) or robotic (*n* = 17) MAL release. Most common preoperative symptoms were postprandial abdominal pain (90%), weight loss (45%), diarrhea (30%), and nausea (25%). Comparing laparoscopic and robotic surgery, neither the median duration of surgery (minutes: 98 (90–290) vs. 125 (80–254); *p* = 0.765), the median length of hospital stay (days: 4 (3–4) vs. 5 (3–6); *p* = 0.179) and intraoperative blood loss (< 50 ml in both groups, *p* = 1.0) showed significant differences. Peak systolic velocity in the CA was significantly reduced postoperatively (cm/s: 320 (200–765) vs. 167 (100–500), *p* < 0.001). Postoperatively, 17 (85%) patients reported symptom improvement, while 4 (20%) patients had no symptom relief at last follow-up. In 3 cases, follow-up imaging showed evidence of respiratory-related CA stenosis.

**Conclusion:**

Despite being complex and challenging procedures, laparoscopic and robotic-assisted MAL release are safe procedures with low risk of postoperative complications and good longterm outcomes.

## Introduction

Median arcuate ligament syndrome (MALS) describes a rare disorder that is defined by the association of unspecific but typical clinical symptoms with external compression of the celiac artery (CA) by the median arcuate ligament (MAL), including (postprandial) abdominal pain, nausea, diarrhea, and weight loss [1, 2]. The anatomical correlate is a variant of the celiac artery with a cranial origin or a caudal insertion of the MAL resulting in a respiratory-dependent obstruction of the celiac blood flow causing chronic mesenteric ischemia with its respective symptoms [1]. However, the exact underlying pathomechanisms remain not entirely understood and compression of the CA is a common finding also in non-symptomatic individuals [3]. While other common causes of gastrointestinal symptoms should first be excluded, duplex ultrasonography (DUS) is the imaging modality of choice for diagnosis of MALS evaluating peak systolic velocities (PSV) during expiration [4]. Additional imaging including CT or MR angiography can further help to identify mesenteric stenosis in patients who would benefit from surgical therapy and to exclude any other underlying diseases [1, 4]. After the first description of surgical decompression of the celiac artery by Dunbar et al. [5], various surgical approaches for MAL release have been described including open and minimally invasive procedures [6, 7]. Today, laparoscopic median arcuate ligament release is widely used. However, with the introduction of robotic systems, improved access to the ligament is now possible including a greater range of instrument motion, better visualization with high-resolution three-dimensional optics and precise preparation due to tremor filtering [8–10]. Given the rarity of the disease, data about perioperative outcome and therapy success of the surgery remains limited to inconsistent. We therefore aimed at evaluation of the postoperative complication rate and outcome of complaints after minimally invasive MAL release investigating differences between laparoscopic and robotic-assisted approach.

## Materials and methods

A retrospective analysis of all patients who underwent minimally invasive surgery for MALS at the Department of Surgery, CCM| CVK of the Charité – Universitätsmedizin Berlin from January 2014 to October 2023 was performed. Clinical charts were reviewed for demographic and clinical data including presentation of symptoms (multiple responses possible), radiological imaging, and perioperative data. MALS diagnosis was based on radiological proof of compression of the celiac artery either via DUS (showing expiratory PSVs greater than 200 cm/s), CT or MR angiography. All patients received esophagoduodenoscopy and an additional abdominal imaging (ultrasound, CT or MRI) prior to indication for surgery to rule out other abdominal pathologies. Follow-up assessment included evaluation of clinical symptoms (multiple responses possible) and additional radiological imaging in case of persistent or recurrent symptoms. The first obligatory follow-up was performed approximately 4 to 6 weeks after surgery and longterm follow-up data (longer than 12 months) was available for *n* = 11 patients. In most cases and mandatorily when patients still presented with symptoms, DUS of the celiac artery was performed. Symptomatic patients were reassessed after 3 to 6 months postoperatively with additional DUS or cross-sectional imaging for evaluation of possible interventional treatment. 12 to 15 months postoperatively and at the end of study date symptom relief of patients was reassessed. Exclusion criteria were visceral resections (e.g. liver, pancreas) in addition to MALS release and surgery for recurrent disease. The study was approved by the local Institutional Ethics Committee of Charité – Universitätsmedizin Berlin (EA2/107/24) and was conducted in accordance with the Declaration of Helsinki.

### Operative technique

All procedures were performed under general anesthesia and patients were placed in supine position with moderate reverse Trendelenburg position. For laparoscopic MAL release, the optical trocar was placed supraumbilically, two 12-mm trocars were placed in the left and right lower abdomen, one 5-mm trocar subxyphoidal and another 5-mm trocar was placed in the left upper abdomen. Exposure of the proximal stomach was achieved by retraction of the left hepatic lobe. The lesser sac was opened via the lesser omentum and the common hepatic artery was identified and dissected. Next, the right diaphragmatic crus was dissected achieving exposure of the supraceliac aorta. After identification of the median arcuate ligament, it was thoroughly divided down to the origin of the celiac trunk achieving its complete liberation. The procedure was terminated when no remaining muscle or nerve fibers crossing the celiac trunk were identified. For robotic MAL release, one 12-mm optical trocar was positioned umbilical followed by another 12-mm trocar in the right mid-abdomen. Three 8-mm trocars were placed in the upper abdomen with a 7-cm spacing starting below the right costal arch. The following dissection steps were concordant to the laparoscopic approach resulting in complete dissection of the celiac artery.

### Statistical analysis

Continuous variables are displayed as medians (range), categorical variables as frequencies. Mann-Whitney-U test was used for group comparison of metric variables, the Fisher’s exact test for categorical variables. The significance level was set to 0.05. Statistical analyses were performed using SPSS Statistics software, version 27 (IBM Armonk, NY, USA).

## Results

A total of 20 patients underwent minimally invasive MAL release within the study period, thereof *n* = 3 laparoscopic and *n* = 17 robotic-assisted. The respective preoperative characteristics are demonstrated in Table 1. Most patients were female (*n* = 14, 67%), median age was 35 years and median BMI 21.8 kg/m^2^. When comparing patients regarding the minimally invasive approach for MAL release, no significant differences in terms of the preoperative characteristics were found.

**Table 1:** Preoperative characteristics of patients undergoing laparoscopic and robotic-assisted MAL release

		Total (*n* = 20)	Laparoscopic (*n* = 3)	Robotic-assisted (*n* = 17)	*p* value
Gender ratio	Female: Male	14:6	2:1	12:5	1.0
Age^1^		35 (12–72)	36 (19–47)	34 (12–72)	0.765
BMI [kg/m^2^]^1^		21.8 (14.2–34.1)	21.5 (18.4–22.6)	22.0 (14.2–34.1)	0.712
ASA	1	5 (25%)	1 (33%)	4 (23%)	1.0
	2	15 (75%)	2 (67%)	13 (77%)	
Comorbidities	Hypertension	4 (20%)	0 (0%)	4 (23%)	1.0
	GERD	4 (20%)	0 (0%)	4 (23%)	1.0
	Depression	4 (20%)	0 (0%)	4 (23%)	1.0
	Atrial fibrillation	1 (5%)	0 (0%)	1 (6%)	1.0
	IBS	1 (5%)	0 (0%)	1 (6%)	1.0
Prior abdominal surgery		6 (30%)	2 (67%)	4 (23%)	0.202
Expiratory PSV [cm/s]^1^		320 (200–765)	315 (200–370)	320 (200–765)	0.941
Additional imaging	CT	12 (80%)	2 (67%)	10 (59%)	1.0
	MRI	9 (45%)	1 (33%)	8 (47%)	1.0
SMA stenosis		3 (15%)	0 (0%)	3 (18%)	1.0

Values in parentheses are percentages unless indicated otherwise; ^1^median (range); MAL, Median arcuate ligament; BMI, Body mass index; ASA, American Society of Anesthesiologists classification; GERD, Gastroesophageal reflux disease; IBS; Irritable bowel syndrome; PSV, peak systolic velocity; SMA, Superior mesenteric artery

**Fig. 1:**
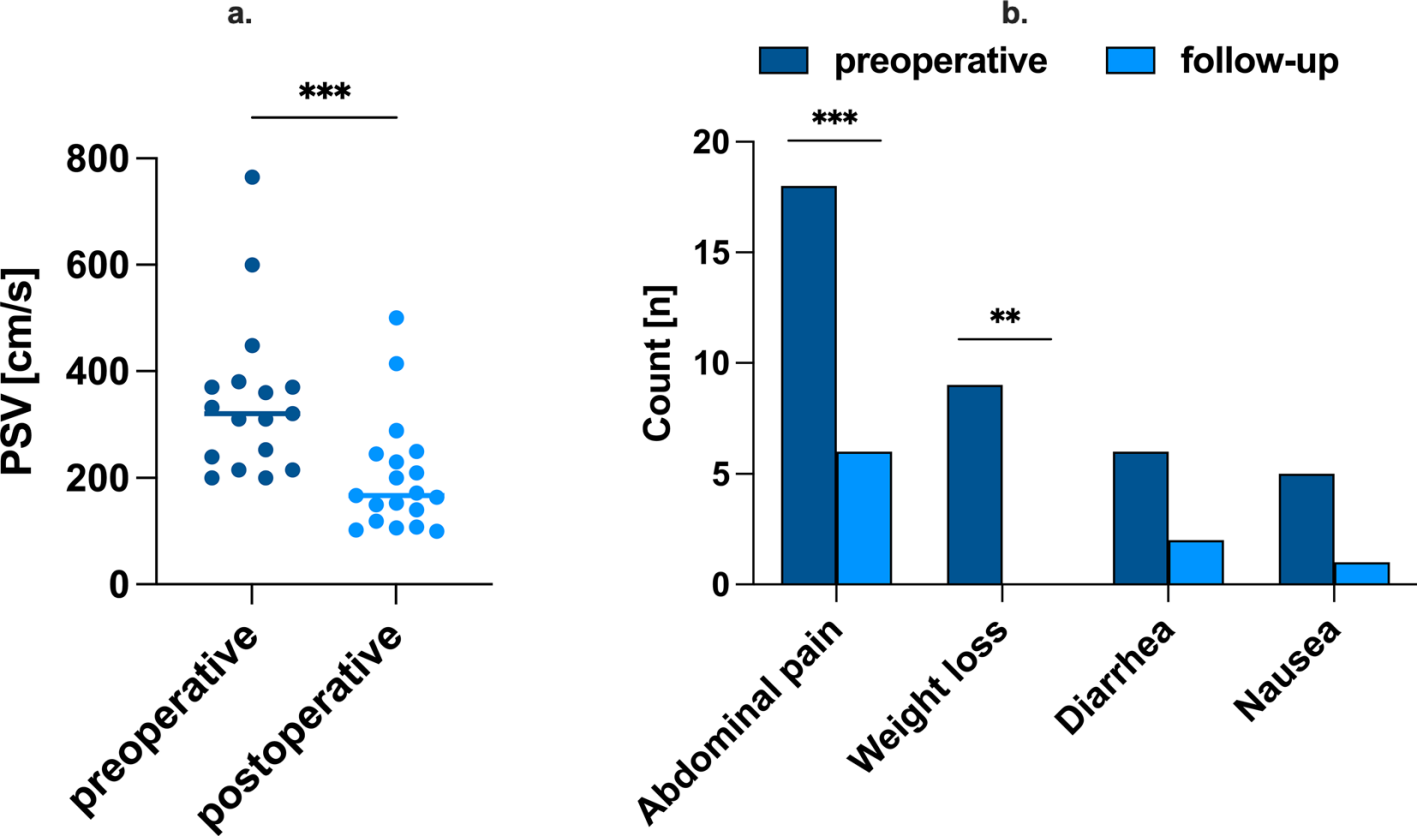
Surgical release of MAL significantly reduces expiratory PSV and leads to symptom relief. **a**. Preoperative expiratory PSV in the celiac artery was significantly higher than postoperative expiratory PSV with 320 cm/s (200–765) vs. 167 (100–500) cm/s, p<0.001. Group values were compared using Mann-Whitney U test. **b**. Symptoms before and at follow-up after surgical MAL release in the total cohort (multiple responses possible). Group values were compared using Fisher’s exact test. MAL, median arcuate ligament; PSV, peak systolic velocity

The perioperative details of the study cohort are shown in Table 2. Median duration of surgery was 124 min and median length of hospital stay 4 days. No conversions to open surgery had to be pursued, the intraoperative blood loss was less than 50 ml and only one minor complication (wound infection, SSI 1) occurred. 17 (85%) patients experienced postoperative symptom relief and MAL release led to a significant reduction in median expiratory PSV (see Fig. 1a). In five patients, a histological specimen of tissue around the celiac artery was assembled. In three of the five cases, activated lymph nodes without evidence of malignancy were proven, one sample showed ganglionic tissue and one sample fibrotic tissue. Again, no significant differences regarding perioperative characteristics were found in terms of surgical approach for MAL release.

**Table 2:** Perioperative characteristics of patients undergoing laparoscopic and robotic-assisted MAL release

		Total (*n* = 20)	Laparoscopic (*n* = 3)	Robotic-assisted (*n* = 17)	*p* value
Duration of surgery [min.]^1^		124 (80–290)	98 (90–290)	125 (80–254)	0.765
Conversion		0 (0%)	0 (0%)	0 (0%)	1.0
Blood loss [ml]^1^		< 50	< 50	< 50	1.0
Transfusion		0 (0%)	0 (0%)	0 (0%)	1.0
ICU		1 (5%)	0 (0%)	1 (6%)	1.0
Length of hospital stay [days]^1^		4 (3–6)	4 (3–4)	4 (3–6)	0.179
Minor complications		1 (5%)	0 (0%)	1 (6%)	1.0
Major complications		0 (0%)	0 (0%)	0 (0%)	1.0
Expiratory PSV [cm/s]^1^		167 (100–500)	147 (140–153)	172 (100–500)	0.491
Postoperative symptom relief		17 (85%)	3 (100%)	14 (82%)	1.0
Histological specimen		5 (25%)	1 (33%)	4 (23%)	1.0
Lymph nodes		3 (60%)	0 (0%)	3 (75%)	
Ganglionic tissue		1 (20%)	0 (0%)	1 (25%)	
Fibrotic tissue		1 (20%)	1 (100%)	0 (0%)	

Values in parentheses are percentages unless indicated otherwise; ^1^median (range); MAL, Median arcuate ligament; PSV, peak systolic velocity

After a median follow-up of 14.5 months (1.5–113 months), 55% (*n* = 11) of all patients stated complete symptom relief while 25% (*n* = 5) described partial symptom relief. However, *n* = 4 patients did not benefit from the surgical therapy (see Table 3). Most common preoperative symptom was abdominal pain in *n* = 18 (90%) patients, followed by unintentional weight loss in *n* = 9 (45%) patients (Table 4). At follow-up, most common residual symptoms were abdominal pain (*n* = 6, 30%) and diarrhea (*n* = 2, 10%). In *n* = 3 patients, radiological imaging revealed a recurrence or persistent stenosis of the celiac artery, and one patient received stenting of the celiac artery. However, this intervention did not achieve symptom relief either. No differences between follow-up characteristics were identified between laparoscopic and robotic-assisted patients.

**Table 3:** Characteristics of patients undergoing laparoscopic and robotic-assisted MAL release at follow-up

			Total (*n* = 20)	Laparoscopic (*n* = 3)	Robotic-assisted (*n* = 17)	*p* value
Symptom relief	Complete		11 (55%)	2 (67%)	9 (54%)	1.0
	Partial		5 (25%)	1 (33%)	4 (23%)	
	None		4 (20%)	0 (0%)	4 (23%)	
Imaging follow up	DUS		8 (40%)	1 (33%)	7 (41%)	1.0
	CTA		4 (20%)	1 (33%)	3 (18%)	
	MRA		1 (5%)	0 (0%)	1 (6%)	
Stenosis			3 (15%)	0 (0%)	3 (27%)	1.0

Values in parentheses are percentages unless indicated otherwise; DUS, duplex ultrasonography; CT, computed tomography angiography; MRA, magnetic resonance angiography

**Table 4:** Symptoms of patients undergoing laparoscopic and robotic-assisted MAL release preoperatively and at follow-up

			Total (*n* = 20)	Laparoscopic (*n* = 3)	Robotic-assisted (*n* = 17)
Preoperative	Abdominal pain		18 (90.0%)	3 (100%)	15 (88%)
	Weight loss		9 (45.0%)	1 (33%)	8 (47%)
	Diarrhea		6 (30.0%)	0 (0%)	6 (35%)
	Nausea		5 (25.0%)	1 (33%)	4 (23%)
Postoperative	Abdominal pain		6 (30%)	1 (33%)	5 (29%)
	Weight loss		0 (0%)	0 (0%)	0 (0%)
	Diarrhea		2 (10%)	0 (0%)	2 (12%)
	Nausea		1 (5%)	0 (0%)	1 (6%)

Values in parentheses are percentages unless indicated otherwise

## Discussion

The results of our study demonstrate that minimally invasive release of the MAL is a safe procedure with a low risk of peri- and postoperative complications and a good longterm outcome. The burden of suffering in patients with MALS is high and justifies this technically challenging procedure. In addition, we did not observe any differences between the laparoscopic and robotic-assisted approaches regarding peri- and postoperative outcomes.

Surgical release of the median arcuate ligament requires a precise dissection in the immediate vicinity of the abdominal aorta. The significant potential for damage to the aorta, celiac trunk or hepatic artery with subsequent large volume bleeding demands a high level of surgical expertise and dexterity. While the laparoscopic technique is limited to two-dimensional imaging and a restricted range of motion of the instruments, robotic-assisted surgery offers the advantages three-dimensional visualization, articulated instruments, and higher precision. Thus, it is not surprising, that the robotic-assisted approach of MAL release has become more and more the approach of choice, which is also the case in our center [8–13]: the technical advantages of the robotic system combined with the positive side effect of increased surgeons comfort mainly led to the almost exclusive use of the robotic approach in recent years. Therefore, an even greater shift towards the preference of robotic-assisted MAL release can be expected wherever the robotic systems are available.

Immediate symptom relief was noted in 17 out of 20 patients following surgery. However, at the last follow-up, four patients experienced complete symptom recurrence. As a result, the current study demonstrates an overall success rate of 80%, comprising complete or partial relief, for MAL release. Reports about the success of surgical therapy of MALS vary from 50% to up to 90% [2, 13, 14]. Clear predictive factors for symptom relief from surgery are still lacking, although they are of utmost importance for patient selection. In this regard, Brody et al. developed a predictive model for symptom improvement after laparoscopic MAL release that included expiratory velocity in the CA and age [15]. In their study including 42 patients, 74% had a good, 14% a neutral and 12% a poor outcome measured by SF-36 scores. In another study, Woestemeier et al. demonstrated symptom relief in 12 out of 20 patients after laparoscopic MAL release. They further showed an association between the co-existence of mast cell activation syndrome and incomplete symptom relief [16]. However, the effect of other variables including comorbidities or duration of symptoms remains unclear and needs further investigation.

In the largest series to date, encompassing 74 patients who underwent robotic MAL release, Gerull et al. report promising results with 90% of patients experiencing symptom relief at the 1-year follow-up [13]. Their study promote a rigorous diagnostic and follow-up management: prior to surgery, all patients underwent esophagogastroduodenoscopy, cross-sectional imaging, and right upper quadrant abdominal ultrasound, particularly if the gallbladder was retained, to rule out alternative pathologies. Moreover, if clinical symptoms indicated gastroparesis, an additional gastric emptying study was performed. In case of exclusion of other pathologies, DUS of the celiac artery was performed and an expiratory PSV threshold of higher than 350 cm/s as well as a respiratory variation of at least 50% difference between in- and expiratory PSV were used as eligibility criteria for surgical MAL release. For the current study, the generally accepted PSV threshold of 200 cm/s was chosen to identify patients eligible for surgery after exclusion of other gastrointestinal pathologies [8, 15, 17]. Interestingly, it was noted that three out of the four patients who did not experience postoperative symptom improvement had expiratory peak systolic velocities (PSVs) below the threshold of 350 cm/s. On the other hand, this was also the case in four of the 11 patients with complete symptom relief. Due to the lack of robust guidelines and inconsistencies in diagnostic algorithms for MALS, patient selection for surgical treatment remains challenging. Although there is no explicit threshold, expiratory PSVs greater than 200 cm/s are often considered suggestive of MALS [8, 17], while e.g. Gruber et al. proposed an expiratory PSV greater than 350 cm/s and a deflection angle of the celiac artery higher than 50° as reliable diagnostic tools [18]. Others even suggest a combination of PSVs during breathing maneuvers [19] underlining the lack of consistent diagnostic guidelines.

Similar to diagnostic inconsistencies, there are no guidelines that define follow-up of MAL release patients. In the present study, most patients and particularly when patients continued to present with symptoms, Doppler ultrasound (DUS) of the celiac artery was routinely performed. Moreover, most patients, including all symptomatic individuals, underwent reevaluation three to six months postoperatively, incorporating additional DUS or cross-sectional imaging to assess the potential need for interventional treatment. However, longterm follow-up (one year and beyond) was only feasible for 11 out of the 20 patients enrolled in our study due to loss of follow-up or patient relocation, as well as the predefined study period ending in October 2023. Gerull et al. presented 1-year follow-up data for 62 out of 74 patients, resulting in a longterm follow-up for nearly 84%. Interestingly, in their study, five patients underwent interventional angioplasty and three of those five experienced symptom relief afterwards [13]. In our study, one patient received interventional stenting of the celiac artery. However, symptom relief could not be achieved either. This again highlights the need for additional identification of predictive factors for postoperative outcome.

In the here-presented cohort, no conversion to open surgery was necessary, no relevant blood loss was reported and only one minor complication (Clavien Dindo I, wound infection, SSI 1) was present, demonstrating that both, laparoscopic and robotic-assisted MAL releases are safe procedures. With a median of four days, we presented longer length of hospital stay than recent reports: e.g. Shin et al. showed a mean hospital stay of only one day [8]. Similarly, Gerull et al. reported a length of stay of 0.8 days [13]. However, it should be noted that both studies were conducted in the USA, with different reimbursement systems and infrastructure than in Germany. Consistent with this, in a recent study from another German center, the mean length of hospital stay of 8.3 days was considerably longer than our reported data [16].

Our reported findings provide valuable insight into a procedure that has been poorly described thus far. Due to the rarity of the disease and the subsequent low number of surgical MAL release procedures performed, there are few studies reporting larger series. However, our study has some limitations. As we report a single-center experience, the study cohort still remains limited. Nonetheless, our results are consistent with the existing literature reporting similar studies. In addition, follow-up longer than 12 months was only available for 11 patients due to loss of follow-up or patient relocation. Further studies are needed investigating longterm results in MALS patients that underwent surgery to identify possible risk factors for recurrence or incomplete pain relief. Lastly, only 3 patients underwent laparoscopic MAL release compared to 17 robotic-assisted procedures. Thus, comparisons can only be interpreted with caution. However, as good results were obtained with the robotic-assisted approach, it offers a suitable alternative to the laparoscopic MAL release where available.

## Conclusion

Both, laparoscopic and robotic-assisted release of the median arcuate ligament are safe surgical procedures with low risk of postoperative complications and good peri- and postoperative outcomes for this complex and challenging surgery. Furthermore, additional studies are required to refine the identification of patients who would benefit most from MAL release.

## Data Availability

The data are only available upon reasonable request from the corresponding author and are not openly available for reasons of sensitivity.
